# Mapping the journey: enhancing abortion care in Uganda's health systems

**DOI:** 10.3389/frph.2025.1609662

**Published:** 2025-07-11

**Authors:** Steve Biko Sigu, Isaac Milton Odongo, Wilbert Nango, Silvester Ochieno Okechi, Annah Kukundakwe, John Musoba Kitui

**Affiliations:** ^1^ Department of Quality of Care, Ipas Africa Alliance, Nairobi, Kenya; ^2^ Department of Quality of Care, Ipas Africa Alliance, Kampala, Uganda

**Keywords:** journey mapping, comprehensive abortion care, health systems, post abortion care, client exit interview

## Abstract

**Introduction:**

This study explores women's experiences with abortion care in Uganda's public health system, assessing satisfaction levels and their influence on the uptake of abortion and post-abortion contraception services. Conducted across nine districts, it highlights challenges within a legally restricted abortion landscape that continues to contribute to high rates of unintended pregnancies and unsafe abortions.

**Methods:**

The study employed a mixed-methods approach involving client exit interviews and in-depth interviews across nine Ugandan districts from January to June 2024 in 13 Ipas Alliance-supported health facilities. Data from 440 women aged 15–49 captured demographics, treatment outcomes, abortion service uptake, post-abortion contraception use, and satisfaction levels. Additionally, 63 in-depth interviews provided qualitative insights into women's care experiences. Quantitative data were analyzed using logistic regression to assess associations between sociodemographic factors, provider interaction, and satisfaction with abortion services, offering a comprehensive understanding of the client journey and care quality.

**Results:**

Logistic regression analysis of client exit interview data revealed no statistically significant differences in satisfaction levels across sociodemographic groups, including age, marital status, facility location, education level, or type of abortion service. However, positive provider engagement was strongly associated with higher satisfaction. Clients who experienced respectful and supportive interactions were significantly more likely to report satisfaction (OR: 0.47, 95% CI: 1.14–14.47, *p* = 0.01), and indicated greater willingness to recommend the facility and return for future services, underscoring the importance of quality provider-client engagement. Qualitative insights offered an in-depth understanding of women's experiences with abortion care services across different levels of the health system.

**Conclusion:**

Client satisfaction with abortion services was strongly linked to positive provider engagement rather than sociodemographic factors. Respectful, supportive interactions significantly increased the likelihood of satisfaction with care, future service use of services, and facility recommendation. These findings underscore the critical role of provider-client relationships in improving service experience. To enhance the quality of abortion care, health systems should prioritize respectful counseling, strengthen referral pathways, and address structural constraints to service delivery.

## 1 Introduction

Journey Mapping is a patient-centric activity that details a patient's progress through a healthcare system for a given service (1). Patient journey mapping visualizes the steps, interactions, and experiences a patient goes through when utilizing healthcare services through formal health systems (2). The patient journey comprises five key stages that reflect interactions with the health system: awareness, information sharing, decision-making, engagement, and retention. These stages capture the progression from recognizing a health issue to actively seeking care and maintaining long-term involvement to manage or prevent future health needs. By documenting every stage of the patient's experience with care; from initial symptoms recognition and initial care seeking behavior, through diagnosis, treatment, and follow-up, health systems can gain a comprehensive understanding of the patient's perspective and develop responsive programs to mitigate public health need (3). This method exposes the key touchpoints where patients access information, or face potential barriers, such as delays, confusion, or emotional distress. Mapping the journey not only highlights clinical processes but also uncovers unmet health needs, emotional challenges, and opportunities for improved health systems response, better patient engagement and disease control (4).

Globally, patient journey mapping has been utilized by governments to shape health system-strengthening initiatives and improve access to public health services (5). Patient journey mapping provides valuable insights into the patient experience, enriching learning health systems and enabling holistic care while helping governments identify inefficiencies and gaps that may compromise care quality. By analyzing patient journeys and involving patients in the mapping process, healthcare workers can redesign workflows to reduce waiting time, improve care coordination in high volume public facilities, and personalize support to clients—fostering empathy, shared decision-making, and ultimately driving continuous improvement, greater patient satisfaction, and better health outcomes (6).

Although journey mapping is an emerging approach in exploratory research, it has been effectively used to enhance the uptake and continuity of care across various health conditions such as HIV/AIDS, Tuberculosis, cancer and other chronic conditions. Holt in their study (7), mapping viral hepatitis services across two low- and middle-income countries in the Asia-Pacific region, noted that effective journey mapping should incorporate both providers and clients to effectively understand health systems inefficiencies. The study employed a journey mapping approach using in-depth interviews and focus group discussions with both clients and healthcare providers. The insights generated were instrumental in understanding both the demand and supply sides of the health system, contributing to the design of more responsive and effective programs for the population. Similarly, Pettit's study in the Southern United States (8), used journey mapping to inform the implementation of rapid linkages to HIV programs. Through participatory process mapping, the study engaged HIV patients to identify both modifiable and systemic barriers to care. Ravics study in rural Uganda (9), employed intervention mapping approach to design a chronic care program integrating HIV services with hypertension and diabetes care at accessible community sites. The approach was effective in reducing stigma associated with HIV treatment and improved overall access to care.

Empirical evidence shows that journey mapping is an inclusive and efficient method for engaging both clients and healthcare providers in the intervention planning process, underscoring its value as a structured, method-driven approach to improving health outcomes. The study attempted to utilize journey mapping to strengthen health systems delivery of comprehensive abortion care. An abortion is the ending of a pregnancy before the embryo or fetus reaches viability or can survive outside the uterus (10). Globally, Safe abortion is a simple health care intervention that is safely and effectively delivered by a wide range of health workers using medication or a surgical procedure (11). Due to the unmet need for contraception, an estimated 121 million unintended pregnancies occur globally each year, with 60% ending in abortion—placing a disproportionate burden on low- and middle-income countries (12). Although the overall rate of unintended pregnancies has declined over the past decade, the proportion ending in abortion has risen significantly (13). In many low- and middle-income countries, legal and cultural restrictions force women to seek abortion services covertly, often leading them to unqualified providers who lack the necessary skills to ensure safe care.

Uganda recognizes access to sexual reproductive health including improving access to safe abortion as a fundamental right and has developed a national sexual reproductive health guidelines and policies to strengthen her health systems in fulfilling this right to her citizens. However, Abortion in Uganda is legally restricted, permitted only to save a woman's life (14). Despite the legal restrictions Uganda has an estimated 314,304 induced abortion cases annually with a national abortion rate of 39 per 1,000 women aged 15–49 years old (15). Furthermore, Uganda faces a high burden of abortion-related complications, largely due to inequalities in the health system's preparedness to deliver comprehensive abortion care. The lack of readiness to provide safe and accessible services contributes significantly to the incidence of unsafe abortions, which account for a substantial proportion of maternal deaths (16). These statistics demonstrate a significant regional variation in abortion rates across age cohort and geographies in Uganda. Additionally, Uganda experiences a high total fertility rate at 5.2 livebirth per woman, modern contraceptive prevalence rate of 38% and pregnancy related mortality ratio is 228 as the potential predictors of maternal mortalities related to unsafe abortion (17). Further, most pregnancies in Uganda remain unintended, highlighting the need for improved contraceptive services and access to comprehensive abortion care (18).

Despite legal restrictions and health system inefficiencies, Uganda has made slow but steady progress in transitioning from unsafe to safer abortion services (19). With support and collaboration from stakeholders in the sustainable abortion ecosystem, the country has adopted various strategies including harm reduction, health system strengthening, and abortion stigma reduction to improve its response to safe abortion and post-abortion care. These efforts have contributed to a notable decline in maternal mortality, with the maternal mortality ratio (MMR) decreasing from 336 per 100,000 live births reported in 2016 to 189 per 100,000 in 2022 highlighting a 44% reduction (20). However, to further address the persistent burden of maternal deaths due to unsafe abortion, there is a need for innovative, client-centered approaches that meet women where they are (21). A holistic strategy that addresses the needs of both women, and the health system is essential for sustaining progress and improving health outcomes. Approaches such as client journey mapping, integration of comprehensive abortion care into health systems, and harm reduction strategies have been effectively employed to expand access to safe abortion services (22, 23) Client journey mapping is particularly valuable in visualizing patient experiences, helping to identify key enablers and barriers to comprehensive abortion care (24).

The objective of the study was to explore clients' experiences with abortion care in public health systems and identify the factors associated with high levels of satisfaction. The study employed a journey mapping approach to retrospectively document client experiences with abortion care within Uganda's health system. The study hypothesized that clients' willingness to return to the facility, receipt of personalized care, and overall positive interactions with healthcare workers are key predictors of high satisfaction with abortion care**.** We traced individuals as they accessed, utilized, and exited health facilities, capturing a comprehensive view of their interactions with abortion services and healthcare providers in 9 districts in Uganda. We further analyzed whether satisfaction levels varied across sociodemographic groups, including age, marital status, education level, and the location of the facility where abortion services were obtained.

## 2 Methods

### 2.1 Study design and data sources

The study employed a mixed-methods research design integrating both survey and in-depth interviews to retrospectively document women's experiences with abortion care and identify key factors associated with client satisfaction across nine districts in Uganda. The mixed-methods approach allowed for a comprehensive understanding of both the quantitative patterns and qualitative nuances of abortion care experiences within public health facilities in Uganda. The survey data was obtained from client exit interviews while the qualitative data was obtained from the in-depth interviews. The client exit interviews were conducted first, targeting 440 women then a sample of 63 women were purposefully recruited from the survey participants for in-depth interviews as guided by Palinkas (25), in purposive sampling for qualitative data collection in mixed method research. Quantitative data were collected through the Ipas standard client exit surveys (26), administered to individuals 1–3 days after receiving abortion or abortion related services. The 1–3-day interval allowed participating women, particularly those who had undergone medication abortion, sufficient time to complete the abortion process prior to the interview. The survey was conducted in 13 health facilities across the 9 districts in Uganda in January to April 2024. The survey captured sociodemographic information of the participants, service utilization patterns, perceived quality of care, and overall satisfaction with the services received. Key indicators assessed included waiting time, privacy, provider communication, facility cleanliness, and willingness to return to the facility for future care. The survey data was analyzed to determine variations in satisfaction levels across sociodemographic groups such as age, marital status, education, and location of facility where service was obtained.

To complement and deepen the survey findings, in-depth interviews (IDIs) were conducted with a purposive sub-sample of 63 women in the month February 2024–May 2024. These interviews explored clients' experiences in greater detail, including their pathways to care, interactions with healthcare workers, perceived barriers or facilitators to receiving quality care, and suggestions for service improvement. The IDIs provided rich narrative data that contextualized quantitative trends and offered deeper insight into the personal and systemic factors influencing satisfaction. In-depth interviews (IDIs) were then conducted 3–5 days after the survey to allow for deeper reflection on the care experience. Data from both components were analyzed separately and then integrated during interpretation to generate a holistic understanding of the client journey and satisfaction with abortion care. This design enabled triangulation of findings, increased the validity of results, and supported the development of responsive, client-centered programmatic interventions aimed at improving abortion care services in Uganda's health system.

### 2.2 Study location and settings

The study was conducted in 9 districts in Uganda namely Arua, Tororo, Busia, Iganga, Jinja Mbale, Kampala, Wakisu and Gulu districts between the period January to May 2024. The facilities selected are part of Ipas health systems strengthening ACTUATE project where Ipas is implementing comprehensive abortion care in collaboration with Uganda's Ministry of health. The districts represent varied health care contexts and need which is a typical service delivery environment in which women seek abortion care-related services in Uganda. The selected set of facilities served as sites for both the survey and the qualitative interviews.

### 2.3 Study participants, sampling and sample size

Given the sensitivity surrounding abortion in Uganda, a convenience-based sampling technique was deemed appropriate for this study (27). Facilities implementing the Ipas ACTUATE project were selected based on their monthly caseload and geographic location. Eligibility was limited to facilities with a minimum of 13 comprehensive abortion care cases per month, ensuring the sample size could be attained within the study timeframe. The 46 participating health facilities were first stratified by district, and within each district, further grouped according to abortion service caseloads using 2023 service delivery records. One or two facilities were then randomly selected from each stratum, yielding a final sample of 13 health facilities (5 general hospitals and 8 health centers). This approach ensured balanced representation across districts and service volumes, allowing the study to capture diverse client experiences with abortion care in both urban and rural settings. For the survey participant recruitment, the 13 selected health facilities across 9 districts served as the primary sampling units (28) as guided by Carter's study on a simulation study. Formal consent for study implementation was obtained from both the respective District Health Offices and the medical superintendents of each participating facility while participating women provided consent for both survey and IDIs before the interviews as well as publication of this work. Written informed consent was obtained from each eligible woman prior to participation in both the quantitative survey and the in-depth interviews (IDIs). All interviews were conducted in person to facilitate the provision of written consent before participation. Following the survey, women were invited to take part in follow-up IDIs conducted 3–5 days later, for which separate consent was also obtained. The study targeted all women of reproductive age (15–49 years of age), women were eligible for participation in the study if they were aged 18–49 years, procured abortion or post abortion care in the intervention facilities and consented to participate in the study. Adolescents aged 15–17 years who had procured abortion or post abortion care in the intervention and consented were included in the study only if they were married, as marriage is considered an indicator of having assumed adult responsibilities and independent decision-making roles. Cumulatively, 760 women of reproductive age were recruited at the point of care with 440 consenting to participate in the study representing 58% response rate. For the IDIs, participants were recruited from the pool of 440 to validate journeys based on their experiences with comprehensive abortion care in public facilities. Following the survey interviews, participants were informed about the opportunity to take part in follow-up in-depth interviews (IDIs). A total of 80 clients were recruited, of whom 63 provided consent and participated in the IDIs conducted 3–5 days after the survey.

### 2.4 Data collection, analysis and processing

Data for this study were collected by Ipas-trained research assistants across nine districts in Uganda between January and May 2024. Ten research assistants were recruited from a pool of experienced personnel previously involved in Ipas-supported research activities. Ten research assistants were trained to administer both the client exit surveys and in-depth interviews (IDIs), following successful participant recruitment and informed consent. The training also included modules on abortion values clarification, ethical considerations, and detailed orientation on the study tools. Survey data were collected using Ipas' standardized Client Exit Interview tool, programmed and administered through Kobo toolbox. The tool captured information on abortion service uptake, type of abortion, related services, post-abortion contraception, client satisfaction, and overall experience with abortion care. IDI data was collected using a journey mapping tool designed to explore women's experiences and interactions with abortion care services at various points within the health system and was recorded for transcription. Both tools were piloted for a day and validated to ensure reliability. The combined use of surveys and IDIs allowed for a comprehensive understanding of women's abortion care journeys, from their initial entry into the health system to the completion of care. Survey data were collected using Ipas-encrypted tablets, and all records were securely stored on password-protected Kobo servers, with access limited to the Ipas data lead only. Participants were linked through unique anonymous IDs with no unique identifies were collected from the participants for both survey and IDIs. Data synchronization was performed only once in day during the data collection period and when secure internet access was available during daily debriefs, minimizing risk of unauthorized access during the study. IDI interviews were recorded and transcribed.

Data from the client exit interviews were reviewed for accuracy and completeness, with close attention to the correct capture of all fields consistency while maintaining participant confidentiality. Missing values were addressed using mean substitution, and log transformations were applied where appropriate. The cleaned dataset was aggregated, coded and imported into Stata version 14 for analysis. Sociodemographic variables were categorized into groups including age, marital status, highest educational attainment, and facility location. Diagnostic tests such as the Breusch-Pagan test for heteroscedasticity, correlation analysis for multicollinearity, and the Shapiro–Wilk test for normality tests revealed no significant data issues. A logistic regression analysis was conducted to estimate the odds ratios for various explanatory variables including sociodemographic factors (age, education, marital status), type of abortion service received, access to private counseling, and overall quality of experience to assess their association with client satisfaction. Additionally, the analysis aimed to determine whether significant differences in satisfaction levels existed across different sociodemographic groups. The results were summarized and presented using descriptive tables and graphs. The journey mapping interviews were audio recorded, transcribed in verbatim, coded and Atlas.ti Version 9 used to conduct thematic content analysis. The thematic content analysis focused on the client's overall experiences and journey with abortion services in health systems. Client satisfaction level was measured both quantitative and qualitatively. In the survey, we included indicators to highlight clients' experiences such as treatment with dignity and respect during visit, providing private and confidential care during abortion services, providing provider listening to the needs of the clients, willingness to return to the facility and willingness to recommend the facility to a friend. Results from the qualitative data analysis were presented in verbatim and insights were used to validate and strengthen insights from the survey.

### 2.5 Table of variables

Table 1 below presents a list of variables used in the study.

**Table 1:** List of variables.

Variable	Variable description
Satisfaction	Women's satisfaction with abortion care, measured by their willingness to recommend the facility to others and their intention to return for future services.
Age	Woman's age at the time of interview in completed years
Highest education	Woman's highest education attainment categorized as primary, secondary and tertiary
Marital status	Woman's marital status categorized as single, married and divorced/separated
Facility location	Location of facility where abortion was obtained from categorized as urban and rural
Abortion type	Abortion method received by woman at the facility categorized as Medication abortion (MA), MVA, and others
Provider interaction	Client and provider interaction at the abortion service delivery room
Autonomy in decision	Woman's decision to end the pregnancy.
Postabortion FP	Woman's post abortion contraception use.

### 2.6 Ethical considerations

Ethical approval for this study was granted by the Ethics Review Committee of the Makerere University School of Public Health, with the approval number SPH-2024-560. Additionally, a research permit was secured from the Uganda National Council for Science and Technology (UNCST) under the approval number HS4137ES. Further authorization was obtained from the district medical officer and the heads of the health facilities to conduct onsite data collection at the respective facilities.

## 3 Results

### 3.1 Demographic characteristics of the study participants

A total of 440 women from nine districts in Uganda participated in the study. The majority were young women aged 20–24 years old (36.36%), followed by young adults aged 25–29 years old (22.05%). Adolescents (10–19 years old), adult women (30–34 years old), and older women (35 years and above) accounted for 12.95%, 16.36%, and 12.27% of the sample, respectively. Most participants were married (65.77%), while 27.05% were single women and 8.18% were divorced or separated. Regarding education attainment, 42.27% had completed secondary education, 39.09% had primary education, and 18.64% had attained tertiary-level education. Just over half (50.45%) accessed abortion services at facilities located in urban areas, while 49.55% visited rural facilities. Most participants expressed satisfaction with the services received and indicated they would return to the facility for similar care. Additionally, 84.5% reported receiving private counseling and described positive interactions with the abortion service delivery team. In terms of the type of abortion services sought across the nine districts, service provision varied. Medication abortion was the most reported method, accounting for 52% (*n* = 230) of cases, followed by manual vacuum aspiration (MVA) at 41% (*n* = 180). A smaller proportion of clients reported undergoing other surgical procedures at 6% (*n* = 24), while 1% (*n* = 6) could not recall the specific method used for pregnancy termination. Participant sociodemographic data is demonstrated in Table 2 below:

**Table 2:** Demographic characteristics of participants and variables.

Demographic Characteristics	Categorization	Respondents	Percentage
Age of women	Adolescents 10–19 Years	57	12.95
	Young women 20–24	160	36.36
	Young Adult women 25–29	97	22.05
	Adult women 30–34 years	72	16.36
	Older Women 35+	54	12.27
Marital status	Single	119	27.05
	Married	285	65.77
	Separated	36	8.18
Highest education status	Primary completed	172	39.09
	Secondary completed	186	42.27
	Tertiary completed	82	18.64
Location of facility	Received services from Rural H/c	222	50.45
	Received services from Urban H/c	218	49.5
Private counselling	No	51	11.59
	Yes	389	88.41
Would recommend	No	22	5
	Yes	418	95
Abortion service type	MVA	180	40.91
	Medication abortion	230	52.27
	other surgical procedure	17	3.86
	Other	7	1.59
	Don't know	6	1.36

### 3.2 Qualitative insights

#### 3.2.1 Uptake of abortion and abortion related services

Based on the qualitative interviews, the study examined women's pathways to accessing abortion services and the nature of abortion care offered within Uganda's health system. Findings indicate that a range of abortion services are available across the nine districts, with post-abortion care in public health facilities generally offered at low or no cost. Due to legal restrictions, many women initially sought care from local clinics and pharmacies upon recognizing pregnancy symptoms. These settings often served as the entry point for initiating the abortion process. However, when complications arose or cases became more complex, women were referred or self-referred to public health facilities for post-abortion care. In some instances, both providers and clients relied on informal referral networks, including previous clients, to discreetly navigate the health system. Referral and linkage pathways from community-level care points to formal health facilities were commonly used, particularly for women presenting with complications or advanced pregnancies. At the facility level, the quality of pre-abortion counseling varied; while some clients received comprehensive information to support informed decision-making, others reported inadequate counseling. For example, IDI participant a stated:

“I came to this facility because I had been to some other facility near home and I was getting worse, so my husband and I decided to come here since its main hospital and its government” (IDI participant, 32 years old Tororo District)

Further, qualitative interviews revealed that health facilities generally provided a standardized package of quality care across the nine districts, in line with Uganda's Comprehensive Post-Abortion Care (PAC) Guidelines. Clients accessing abortion services at intervention facilities reported receiving counseling and informational support to aid decision-making, pain management during the procedure, a range of abortion methods, and post-abortion care, including access to contraception. However, the study also highlighted specific socio-cultural dynamics that influenced women's experiences with care. Married women sometimes reported facing pressure from their spouses to terminate pregnancies, while adolescent girls particularly those in school or living with parents expressed fear of parental discovery, leading to emotional distress and reluctance to continue the pregnancy. For many of these women, the urgent need was to terminate the pregnancy, often with limited attention to post-abortion services such as contraceptive counseling. This challenge was underscored by participants who emphasized the emotional weight and social pressures surrounding abortion decisions as expressed below

“First of all, I told him that I am not married, and I don’t want my parents to abuse me. I told him that I didn’t want to go back home because I didn’t want my parents to know that I was pregnant” (IDI participant, 19 years old Mbale Districts)

The observation reflects the experiences of another IDI participant in the Busia district

The “He did not want the pregnancy, and he wanted me to remove/abort it.” (IDI participant, 37 years in Iganga district.).

Post abortion contraceptive services were generally integrated into the comprehensive abortion care packages offered across health facilities in the nine study districts in Uganda. Most facilities provided a wide range of contraceptive options; however, the uptake following abortion varied across regions and facilities due to both individual-level preferences and system-level factors. Notably, some facilities experienced stockouts of long-acting reversible contraceptives (LARCs) during the study period, contributing to inconsistencies in service availability and method uptake across districts. While all clients received counseling on available contraceptive methods, some women reported negative interactions with healthcare providers during the counseling process. These included poor communication, judgmental attitudes, and the use of harsh language, which created barriers to informed decision-making. In contrast, the influence of supportive family members, particularly husbands and sisters, was cited as a key factor in shaping contraceptive choices, as highlighted by in-depth interview (IDI) participant.

“All the services that I needed were provided to me; for example, I wanted the stomach washed and it was washed, and I wanted family planning services and even that one I received” (IDI participant 27 years old, Kampala District).

#### 3.2.2 Client experience and satisfaction level with abortion services

Although infrequent, some participants reported negative interactions with healthcare providers, particularly during contraceptive counseling. These interactions were primarily characterized by poor provider attitudes and ineffective communication, with several women describing instances where health workers used harsh language or raised their voices. Such experiences created emotional discomfort and inhibited open dialogue. In contrast, many women emphasized the supportive role of family members especially husbands and sisters in influencing their contraceptive decisions. The observation is reflected in insights from in-depth interview (IDI) participants.

“…when you come, the nurses need not shout at you, they should handle women with dignity and educate them peacefully” (IDI, Participant 39 years old, Arua district).

Despite these isolated negative experiences, women across all regions participating in qualitative interviews expressed overall satisfaction with the abortion services received. They appreciated that the abortion procedures and accompanying support services were delivered in a manner that was responsive to their individual needs. High levels of client satisfaction were evident through several indicators: women reporting that they were treated with dignity and respect, women who reported that providers listened to their needs, and women who stated they received private and confidential care. Furthermore, women who indicated they would recommend the facility to others for similar services. The observation is reflected by the IDI participant below.

“For me almost all the services I received were very good, especially the act of cleansing the uterus (MVA). This is because I wouldn’t even think about it in the first place, although I have heard about it before. I had no idea it was necessary, but I was told that since the pregnancy was due by four months, I had to go through the process to avoid problems. I loved this since it would be difficult to find transport to take me to Gulu or any other bigger hospital incase things went wrong.” (IDI participant, 34 years Gulu District)

Additionally, we also examined emergency situations, particularly when an abortion initiated in a pharmacy or clinic resulted in an incomplete procedure. The study found that women valued being informed about their health status and the steps being taken. This observation was expressed by the IDI participant below.

“When I reached the hospital, they left whatever they were doing and attended to me” (IDI participant, 23 years Iganga district).

Overall, women seeking abortion care consistently reported positive experiences with the client-centered service delivery approach. They noted prompt attention from health workers, minimal waiting time even at high-volume service points and respectful engagement throughout all stages of care. These sentiments were echoed in narratives from IDI participants, illustrating the value of responsive and compassionate care in shaping client satisfaction.

“The doctor spoke to me in a calm and humble manner. He explained to me what he was going to do. I didn’t have any questions, I only asked him what was happening to me, and he told me I would be okay. He also told me about the different family planning options. Then I told him I had tried three options, and they treated me badly, he said I should get well first then come back to him and we see what to use. The doctor spoke to me with lots of respect and he never judged me anywhere in our interaction.” (IDI, participant 32 years, Tororo District)

### 3.3 Quantitative insights

#### 3.3.1 Results from multivariate logistic regression analysis

Table 3 presents the results from the logistic regression analysis conducted on the client exit interview data. The analysis found no statistically significant differences in satisfaction levels across sociodemographic groups, including age, marital status, facility location, educational attainment, and type of abortion service received. However, positive provider engagement was significantly associated with higher satisfaction levels. Specifically, clients who reported positive interactions with providers were more likely to express satisfaction with abortion services (OR: 0.47, 95% CI: 1.14–14.47, *p* = 0.01). These clients also indicated a greater likelihood of recommending the facility to others and returning for services in the future.

**Table 3:** Logistic regression results.

Satisfaction	Odds ratio	Std. Err.	z	*P* > z	(95% Conf.Interval)	
Age	0.7902158	0.1769578	−1.05	0.293	0.5094845	1.225633
Highest Education	0.7026118	0.2515182	−0.99	0.324	0.3483418	1.417181
Marital status	0.5318031	0.3016421	−1.11	0.266	0.1749621	1.616433
Facility location	1.305389	0.7369195	0.47	0.637	0.4317363	3.946945
Abortion type	1.850185	0.8229356	1.38	0.167	0.7737717	4.424022
Provider interaction	4.523365	2.639532	2.59	0.01	1.441311	14.19599
Autonomy Decision	0.4705385	0.3028903	−1.17	0.242	0.1332503	1.661583
Postabortion FP	1.550499	0.9198303	0.74	0.46	0.4847276	4.959582
_cons	36.67728	54.74622	2.41	0.016	1.967224	683.8176

## 4 Discussion

This study examined clients' experiences with abortion care in public health facilities across nine districts in Uganda, using both quantitative and qualitative methods to identify factors associated with high levels of satisfaction. The insights generated contribute to understanding how health systems can be strengthened to deliver high-quality, client-centered abortion services.

The qualitative findings provide deeper context to the quantitative results by highlighting the complex pathways women navigate to access abortion services in Uganda. Despite legal and social constraints, a range of abortion-related services is available, with post-abortion care often accessible at low or no cost in public health facilities. Most women begin their care-seeking journey at local clinics or pharmacies, particularly where formal care is limited or stigmatized, and later transition to public facilities when complications arise or for more advanced care. Informal referral networks—often facilitated by former clients or trusted community members play a key role in navigating these transitions. These findings underscore the importance of clear and efficient referral and linkage systems, especially for women presenting with complications or late-stage pregnancies. However, the variability in pre-abortion counseling across facilities reflects gaps in consistent, informed decision-making support. Ultimately, client satisfaction hinges on the quality of interpersonal care—how women are received, informed, and supported throughout the process. This aligns with findings from the SACHA study (29), which emphasize the value of timely care, informed choice, and emotional support in improving abortion care experiences.

Variations in the quality of pre-abortion and contraceptive counseling were evident across facilities and nine districts. While some women received comprehensive information to support informed decision-making, others reported limited guidance, reflecting inconsistencies in service delivery. Although most facilities integrated contraceptive services into abortion care and offered a range of methods, uptake varied due to individual preferences and systemic barriers, including stockouts of long-acting reversible contraceptives (LARCs) and negative provider interactions characterized by judgmental attitudes and poor communication. These findings are partially supported by Gashaye study (30), on the prevalence and determinants of women's satisfaction with the quality of safe abortion services in Northwest Ethiopia, which found that, beyond positive interpersonal interactions with abortion service providers, women who received post-abortion contraception were generally more satisfied with the overall quality of abortion care within the health system. These challenges underscore the need for continuous provider training in respectful, client-centered counseling, particularly in sensitive reproductive health contexts. These findings are further confirmed by Geta study that found that understanding women's expectations and perceptions by providing space for uninterrupted care, provide training for healthcare providers to center care on women's needs could enhance overall satisfaction levels (31). This underscores the central role of provider-client interactions in shaping clients' perceptions of quality care, consistent with previous research indicating that interpersonal aspects of care are critical determinants of patient satisfaction in reproductive health services.

Further, access to abortion and post-abortion services was shaped by women's personal circumstances, such as marital status or age in some circumstances. Adolescents, for example, often feared parental discovery, while some married women faced pressure from spouses highlighting the importance of tailored, context-sensitive counseling approaches. These findings align with prior studies, including the Tibebu study in Ethiopia (32), which linked client satisfaction to respectful and dignified care, and the Jemal K study (33), which emphasized the role of compassionate care in improving service uptake. Together, these insights suggest that addressing both provider-related and socio-cultural barriers is essential to improving the overall client experience and supporting women's autonomy in reproductive health decisions in Uganda.

Quantitative findings from the logistic regression analysis of client exit interviews revealed no statistically significant differences in satisfaction levels across sociodemographic groups, including age, marital status, facility location, educational attainment, or the type of abortion service received. These findings are contradicted Eboigbe study (34) that where both adolescents and older women reported high levels of satisfaction with care based on the individual components of care. However, a key finding was the significant association between positive provider engagement and higher satisfaction levels. Clients who experienced respectful, supportive, and communicative interactions with healthcare providers were more likely to report overall satisfaction, express willingness to return for future services, and recommend the facility to others.

Overall, the findings underscore the importance of investing in provider training, ensuring consistent availability of contraceptive methods, and addressing social stigma to improve client experiences. Strengthening referral systems, standardizing counseling protocols, and promoting client-centered approaches are essential steps toward delivering high-quality, equitable, and respectful abortion care in Uganda's health system.

## 5 Conclusion

While notable progress has been made in expanding abortion services across nine districts in Uganda, several areas still require improvement. Addressing these challenges through structured health system interventions and community engagement can promote equitable and respectful care for all women. This study highlights the importance of exploring women's expectations of abortion services in public facilities to better inform service design and delivery. Strengthening referral systems and standardizing pre-abortion counseling are critical to promoting informed decision-making and ensuring the provision of comprehensive care. Community sensitization efforts can help mitigate socio-cultural barriers and support women's reproductive autonomy. Additionally, improving supply chain management to reduce contraceptive stockouts would enhance post-abortion contraceptive uptake and ensure continuity of care. Training healthcare providers in effective communication and client-centered care is also essential to improving provider-client interactions and increasing service utilization.

## Data Availability

The original contributions presented in the study are included in the article/Supplementary Material, further inquiries can be directed to Steve Biko Sigu at bikos@ipas.org.

## References

[1] Bharatan T Devi R Huang P-H Javed A Jeffers B Lansberg P A methodology for mapping the patient journey for noncommunicable diseases in low-and middle-income countries. J Healthc Leadersh. (2021) 13:35–46. 10.2147/JHL.S288966 33542673 PMC7853412

[2] Bulto LN Davies E Kelly J Hendriks JM. Patient journey mapping: emerging methods for understanding and improving patient experiences of health systems and services. Eur J Cardiovasc Nurs. (2024) 23(4):429–33. 10.1093/eurjcn/zvae012 38306596

[3] Hughes RG. “Section VI: Tools for Quality Improvement and Patient Safety Chapter 44. Tools and Strategies for Quality Improvement and Patient Safety,” (2008).

[4] Davies EL Bulto LN Walsh A Pollock D Langton VM Laing RE Reporting and conducting patient journey mapping research in healthcare: a scoping review. J Adv Nurs. (2023) 79(1):83–100. 10.1111/jan.15479 36330555 PMC10099758

[5] Joseph AL Monkman H Kushniruk A Quintana Y. Exploring patient journey mapping and the learning health system: scoping review. JMIR Hum Factors. (2023) 10:e43966. 10.2196/43966 36848189 PMC10012009

[6] Meyer MA. Mapping the patient journey across the continuum: lessons learned from one patient’s experience. J Patient Exp. (2019) 6(2):103–7. 10.1177/2374373518783763 31218254 PMC6558942

[7] Holt B Mendoza J Nguyen H Doan D Nguyen TH Mercado TB Putting people at the center: methods for patient journey mapping of viral hepatitis services across two LMICs in the Asia Pacific. BMC Health Serv Res. (2025) 25(1):427. 10.1186/s12913-025-12543-w 40128698 PMC11934455

[8] Pettit AC Pichon LC Ahonkhai AA Robinson C Randolph B Gaur A Comprehensive process mapping and qualitative interviews to inform implementation of rapid linkage to HIV care programs in a mid-sized urban setting in the Southern United States. J Acquir Immune Defic Syndr. (2022) 90:S56–64. 10.1097/QAI.0000000000002986 35703756 PMC9204789

[9] Ravicz M Muhongayire B Kamagaju S Klabbers RE Faustin Z Kambugu A Using intervention mapping methodology to design an HIV linkage intervention in a refugee settlement in rural Uganda. AIDS Care. (2022) 34(4):446–58. 10.1080/09540121.2021.1900532 33749418 PMC8452793

[10] Shakhatreh HJM Salih AJ Aldrou KKAR Alazzam FAF Issa MSB. Medico-legal aspects of abortion: updates of the literature. Med Arch. (2022) 76(5):373. 10.5455/medarh.2022.76.373-376 36545456 PMC9760240

[11] Erdman JN. The WHO abortion care guideline: law and policy—past, present, and future. Int J Gynecol Obstet. (2023) 162(3):1119–24. 10.1002/ijgo.15017 37462065

[12] Aragaw FM Amare T Teklu RE Tegegne BA Alem AZ. Magnitude of unintended pregnancy and its determinants among childbearing age women in low and middle-income countries: evidence from 61 low and middle income countries. Front Reprod Health. (2023) 5:1113926. 10.3389/frph.2023.1113926 37533507 PMC10393037

[13] Bearak J Popinchalk A Ganatra B Moller A-B Tunçalp Ö Beavin C Unintended pregnancy and abortion by income, region, and the legal status of abortion: estimates from a comprehensive model for 1990–2019. Lancet Glob Health. (2020) 8(9):e1152–61. 10.1016/S2214-109X(20)30315-6 32710833

[14] Republic Of Uganda. “The Constitution of the Republic of Uganda, 1995. National Objectives and Directive Principles of State Policy. Arrangement of Objectives,” (1995). Available online at: chrome-extension://efaidnbmnnnibpcajpcglclefindmkaj/https://www.ngobureau.go.ug/sites/default/files/laws_regulations/2020/12/Uganda%20Constitution%201995.pdf (Accessed January 30, 2025).

[15] Prada E Atuyambe LM Blades NM Bukenya JN Orach CG Bankole A. Incidence of induced abortion in Uganda, 2013: new estimates since 2003. PLoS One. (2016) 11(11):e0165812. 10.1371/journal.pone.0165812 27802338 PMC5089684

[16] Atuhairwe S Gemzell-Danielsson K Byamugisha J Kaharuza F Tumwesigye NM Hanson C. Abortion-related near-miss morbidity and mortality in 43 health facilities with differences in readiness to provide abortion care in Uganda. BMJ Glob Health. (2021) 6(2):e003274. 10.1136/bmjgh-2020-003274 33547174 PMC7871269

[17] U. Bureau. “GOVERNMENT OF UGANDA Uganda Demographic and Health Survey 2022,” (2023). Available online at: www.ubos.org

[18] Curtis C Huber D Moss-Knight T. “International Perspectives on Sexual and Reproductive Health Global Health Fellows Program,” (2010). 10.1363/ipsrh.36.044.10 20403805

[19] Bankole A Remez L Owolabi O Philbin J Williams P. “From Unsafe to Safe Abortion in Sub-Saharan Africa: Slow but Steady Progress,” (2020). 10.1363/2020.32446.

[20] Birabwa C Banke-Thomas A Waiswa P Semaan A Kananura RM van Olmen J Maternal health in cities: analysis of institutional maternal mortality and health system bottlenecks in Kampala city Uganda, 2016–2021. J Glob Health Rep. (2024) 8:1–14. 10.29392/001c.116248

[21] Singh S Remez L Sedgh G Kwok L Onda T. “Uneven Progress and Unequal Access,” New York, NY 10038 USA, (January 2018).

[22] Kagaha A Manderson L. Power, policy and abortion care in Uganda. Health Policy Plan. (2021) 36(2):187–95. 10.1093/heapol/czaa136 33347559 PMC7996642

[23] Kayondo SP Kaye DK Nabatanzi SL Nassuuna S Musana O Namagembe I Challenges and opportunities from using abortion harm reduction and value clarification and attitude transformation engagements for safe abortion advocacy in Uganda. Reprod Health. (2023) 20(1):97. 10.1186/s12978-023-01637-5 37381001 PMC10303852

[24] Duffy D Mishtal J Grimes L Murphy M Reeves K Chakravarty D Information flow as reproductive governance. Patient journey analysis of information barriers and facilitators to abortion care in the republic of Ireland. SSM Popul Health. (2022) 19:101132. 10.1016/j.ssmph.2022.101132 35711728 PMC9194449

[25] Palinkas LA Horwitz SM Green CA Wisdom JP Duan N Hoagwood K. Purposeful sampling for qualitative data collection and analysis in mixed method implementation research. Adm Policy Ment Health Ment Health Serv Res. (2015) 42(5):533–44. 10.1007/s10488-013-0528-y PMC4012002 24193818

[26] Acre VN Dijkerman S Calhoun LM Speizer IS Poss C Nyamato E. The association of quality contraceptive counseling measures with postabortion contraceptive method acceptance and choice: results from client exit interviews across eight countries. BMC Health Serv Res. (2022) 22(1):1519. 10.1186/s12913-022-08851-0 36514040 PMC9749205

[27] Golzar J Tajik O. “Convenience Sampling,” (2022).

[28] Carter ED Maiga A Do M Sika GL Mosso R Dosso A The effect of sampling health facilities on estimates of effective coverage: a simulation study. Int J Health Geogr. (2022) 21(1):20. 10.1186/s12942-022-00307-2 36528582 PMC9758803

[29] Lewandowska M Scott R Meiksin R Reiter J Salaria N Lohr PA How can patient experience of abortion care be improved? Evidence from the SACHA study. Women’s Health. (2024) 20. 10.1177/17455057241242675 PMC11128172 38794997

[30] Gashaye KT Taddese AA Birhan TY. Prevalence and determinants of women’s satisfaction on the quality of safe abortion service in Northwest Ethiopia. Arch Public Health. (2022) 80(1):146. 10.1186/s13690-022-00897-0 35614476 PMC9134683

[31] Geta T Israel E Kebede C. Client satisfaction with abortion care service and its associated factors among women in Ethiopia: a systematic review and meta-analysis. BMC Womens Health. (2024) 24(1):287. 10.1186/s12905-024-03139-3 38745273 PMC11091994

[32] Tibebu NS Alemu MB Rade BK Kassie BA Bicha MM Mihret MS Women’s satisfaction with comprehensive abortion care services and associated factors in central Gondar zone public primary hospitals, northwest Ethiopia, 2023. Front Reprod Health. (2024) 6:1400359. 10.3389/frph.2024.1400359 39411054 PMC11473510

[33] Jemal K Hailu D Mekonnen M Tesfa B Bekele K Kinati T. The importance of compassion and respectful care for the health workforce: a mixed-methods study. J Public Health. (2021) 31:167–78. 10.1007/s10389-021-01495-0/Published PMC7951938 33728258

[34] Eboigbe E Gadama L Filippi V Mehrtash H Adu-Bonsaffoh K Bello FA Adolescents’ satisfaction with care for abortion-related complications in 11 Sub-Saharan African countries: a cross-sectional facility-based study. International Journal of Gynecology and Obstetrics. (2022) 156(S1):63–70. 10.1002/ijgo.13896 34676896

